# Impact of the COVID-19 pandemic on management of surgically treated laryngeal squamous cell carcinoma

**DOI:** 10.17305/bb.2023.9481

**Published:** 2024-02-01

**Authors:** Alexander Lein, David T Liu, Markus Haas, Almir Salkic, Azra Ibrisevic, Sabrina Uscuplic, Alen Harcinovic, Taria Brkic, Thomas Thurner, Faris F Brkic

**Affiliations:** 1 Department of Otorhinolaryngology, Head and Neck Surgery, Medical University of Vienna, Vienna, Austria; 2 Department of Otorhinolaryngology, University Clinical Center Tuzla, Tuzla, Bosnia and Herzegovina; 3 Friedrich-Alexander University, Erlangen-Nuernberg, Germany

**Keywords:** Coronavirus disease 2019 (COVID-19) pandemic, laryngeal squamous cell carcinoma (LSCC), treatment modality, adjuvant therapy

## Abstract

The coronavirus disease 2019 (COVID-19) pandemic has significantly impacted the treatment of cancer patients, particularly in terms of treatment choices. This study aimed to assess the effects of the COVID-19 pandemic on the management of surgically treated laryngeal squamous cell carcinoma (LSCC) patients, focusing specifically on changes in treatment modalities. We retrospectively analyzed the data from 102 patients who underwent surgical treatment for LSCC between January 1, 2019 and December 31, 2021, at our tertiary medical center. Patient demographics, histological characteristics, and treatment modalities were extracted from electronic medical records and compared between two time periods: pre-COVID-19 and during COVID-19, marked by the introduction of the hospital entry triage. Of the total patients, 53 (52%) were in the pre-COVID-19 group, and 49 (48%) were in the COVID-19 group. No significant differences in patient characteristics at the initial work-up were observed between the two groups. However, a significant shift in treatment modalities was noted. Fewer patients received postoperative adjuvant therapy in the COVID-19 group (70.5%) compared to the pre-COVID-19 group (95.5%). Importantly, this change did not significantly impact the one-year overall survival (OS) rates. The reduction in the use of postoperative adjuvant therapy during the COVID-19 pandemic may be attributed to efforts to minimize hospital visits due to the risk of COVID-19 infection. Further research is warranted to validate these findings and to investigate the potential effects of such changes in treatment modalities on the long-term survival.

## Introduction

The coronavirus disease 2019 (COVID-19) pandemic had an unprecedented impact on healthcare providers around the world. Systemic constraints, case reductions, and diagnostic delays substantially prevented the timely delivery of comprehensive care [1]. These challenges were particularly pronounced in the management of head and neck cancer patients. These patients presented with more advanced forms of the disease [2], and the proximity of these cancers to the respiratory tract implied that examinations and procedures carried an elevated risk of transmission and infection [3]. In fact, a recent study showed that changes in the healthcare system due to the COVID-19 pandemic led to a shift in primary treatment regimens for cancer patients from surgery to radiotherapy and chemotherapy [4]. Similar observations were already noted for head and neck cancer cases in the early stages of the pandemic [5]. These changes in treatment modalities could potentially result in worse short- and long-term survival outcomes [6].

Laryngeal squamous cell carcinoma (LSCC) may be particularly affected by this shift in treatment modalities, due to the complexity of optimal treatment strategies for advanced stages [7, 8]. With an estimated 166,000 new cases annually, LSCC ranks as the second most common head and neck cancer globally [9]. The current treatment standard for LSCC includes surgery and/or radiation therapy, which are chosen based on the clinical stage, the patient’s overall condition, and personal preference. For advanced stages, adjuvant therapy, such as radiation therapy or radio-chemotherapy, is often administered after the primary surgery to lower the risk of recurrence [10]. Notably, postoperative radio-chemotherapy (PORCT) is associated with a significant survival benefit compared to postoperative radiotherapy (PORT) alone [8].

To the best of our knowledge, no studies have yet investigated the impact of the COVID-19 pandemic on the management of LSCC patients. Therefore, the aim of the current study was to analyze the influence of the COVID-19 pandemic on treatment modalities for LSCC patients and to assess its potential impact on short-term survival.

## Materials and methods

### Study population and study design

We included all patients who underwent surgical treatment for LSCC between January 1, 2019 and December 31, 2021 at the Department of Otorhinolaryngology at the University Clinical Center Tuzla, Bosnia and Herzegovina. Data on patient demographics, tumor characteristics, and treatment modalities were retrospectively obtained from electronic medical records. Treatment modalities included primary surgery (pOP), pOP with PORT, pOP with PORCT, and salvage surgery. The types of surgery included in this study were total laryngectomy (TL), TL with subsequent neck dissection (ND), cordectomy (CE), supratracheal partial laryngectomy (STPL), and supraglottic partial laryngectomy (SGPL).

We divided our cohort into two groups based on the initiation of entrance triage on June 21, 2020. The first timeframe, representing the pre-COVID-19 period, ranged from January 1, 2019 to June 20, 2020. Accordingly, the second timeframe, corresponding to the COVID-19 period, ranged from June 21, 2020 to December 31, 2021. Staging was performed in accordance with the 8th edition of the American Joint Committee on Cancer (AJCC) cancer staging manual [11].

### Ethical statement

The study was conducted in accordance with the Declaration of Helsinki and approved by the Ethics Committee of the University Clinical Center Tuzla (Nr. 02-09/2-79/22). Given the retrospective nature of the study, the requirement for patient consent was waived.

### Statistical analysis

All statistical analyses were conducted using GraphPad Prism (Version 9, GraphPad Software, San Diego, CA, USA) and STATA (Version 14, StataCorp LLC, College Station, TX, USA). Due to the size of the patient sample, a normal data distribution was assumed. Descriptive data were presented using mean and standard deviation (SD). To test for significant differences between categorial variables, the Pearson’s chi-squared test was employed. If one or more cell counts were below five, Fisher’s exact test was used. Survival curves were generated using the Kaplan–Meier estimator. The overall survival (OS) was defined as the time from initial treatment to death from any cause. Hazard ratios (HRs) were calculated using univariable Cox-proportional hazard models. Since our model comprised only 12 events, multivariable analysis was omitted to avoid the risk of overfitting [12]. A *P* value of ≤ 0.05 was considered statistically significant. The mean follow-up time was 1.7 ± 1.1 years.

## Results

### Patient characteristics and treatment modalities

A total of 102 patients were included in this study, 53 (52%) in the pre-COVID-19 group and 49 (48%) in the COVID-19 group. Patient demographics and tumor characteristics are presented in Table 1. The mean age of the patients was 62.8 ± 7.64 years in the pre-COVID-19 group and 64.4 ±10.5 years in the COVID-19 group. In the pre-COVID-19 group, 50 patients (94.3%) were male and 3 patients (5.7%) were female, while in the COVID-19 group 44 patients (89.8%) were male and 5 patients (10.2%) were female. There were no statistically significant differences in age (*P* ═ 0.174) or gender (*P* ═ 0.394) between the two study groups. Throughout the observed period, 90 patients (88.2%) presented with an initial cancer diagnosis, while 12 patients (11.8%) had already received prior therapy. No statistically significant difference in initial cancer diagnosis was observed between the pre-COVID-19 group and the COVID-19 group (*P* ═ 0.278).

**Table 1:** Patient demographic characteristics, tumor characteristics, types of surgery, and utilized treatment modalities

**Variables/Categories**	**Total**	** Pre-COVID-19 group**	** COVID-19 group**	***P* value**
		*n* **(%)**	***n*** **(%)**	
*Number of patients*	102	53 (100.0%)	49 (100.0%)	
*Age (years)*				
≥65	55	32 (60.4%)	23 (46.9%)	
<65	47	21 (39.6%)	26 (53.1%)	0.174
*Sex*				
Male	94	50 (94.3%)	44 (89.8%)	
Female	8	3 (5.7%)	5 (10.2%)	0.394
*Initial diagnosis*				
No	12	8 (15.1%)	4 (8.2%)	
Yes	90	45 (84.9%)	45 (91.8%)	0.278
*T stage*				
1-2	77	38 (71.7%)	39 (79.6%)	
3-4	25	15 (28.3%)	10 (20.4%)	0.244
*N stage*				
0	87	47 (88.7%)	40 (81.6%)	
+	15	6 (11.3%)	9 (18.4%)	0.315
*M stage*				
0	100	51 (96.2%)	49 (100.0%)	
+	2	2 (3.8%)	0 (0.0%)	0.268
*Stage*				
I-II	73	35 (66.0%)	38 (77.6%)	
III-IV	29	18 (34.0%)	11 (22.4%)	0.198
*Grade*				
1	8	5 (9.4%)	3 (6.1%)	
2	60	32 (60.4%)	28 (57.1%)	
3	17	6 (11.3%)	11 (22.4%)	0.329
Unknown	17	10 (18.9%)	7 (14.4%)	
*Type of surgery*				
TL	18	10 (18.9%)	8 (16.3%)	
TL+ND	23	11 (20.8%)	12 (24.5%)	
CE	52	26 (49.1%)	26 (53.1%)	
STPL	5	3 (5.7%)	2 (4.1%)	
SGPL	4	3 (5.7%)	1 (2.0%)	0.895
*Treatment modality*				
pOP	15	2 (3.8%)	13 (26.5%)	
pOP + PORT	49	26 (49.1%)	23 (46.9%)	
pOP + PORCT	24	16 (30.2%)	8 (16.3%)	
Salvage surgery	14	9 (17.0%)	5 (10.2%)	**0.007**

Significant results (*P* ≤ 0.05) are highlighted in bold print. Distributional differences between the pre-COVID-19 and the COVID-19 groups were analyzed using either Fisher’s exact test or Pearson’s chi-square test. The pre-COVID-19 period represents the time period prior to the introduction of the entrance triage, while the COVID-19 period represents the time period after the introduction of the entrance triage. COVID-19: Coronavirus disease 2019; TL: Total laryngectomy; ND: Neck dissection; CE: Cordectomy; STPL: Supratracheal partial laryngectomy; SGPL: Supraglottic partial laryngectomy; pOP: Primary surgery; PORT: Postoperative radiotherapy; PORCT: Postoperative radio-chemotherapy.

Regarding the histological characteristics, we observed no statistical differences between T stage (*P* ═ 0.244), N stage (*P* ═ 0.315), M stage (*P* ═ 0.198), and tumor grade (*P* ═ 0.329). In the pre-COVID-19 group, 35 patients (66.0%) presented with stage I-II cancer, while 18 patients (34.0%) presented with stage III-IV cancer. In the COVID-19 group, 38 patients (77.6%) presented with stage I-II cancer, while 11 patients (22.4%) presented with stage III-IV cancer. There was no statistically significant difference between the two study groups (*P* ═ 0.329).

Next, we compared the treatment modalities by examining patients who were primarily treated surgically without postoperative adjuvant treatment (those who underwent only pOP) against the groups of patients who received postoperative adjuvant treatment (those who underwent either pOP with PORT or pOP with PORCT). Significantly fewer patients were treated with pOP and adjuvant therapy in the COVID-19 group (70.5% vs 95.5%) (*P* ═ 0.002).

Moreover, we observed a significant difference in overall treatment modalities between the pre-COVID-19 and the COVID-19 group (*P* ═ 0.007). In particular, the proportion of patients receiving pOP with PORCT was lower in the COVID-19 group (16.3%) compared to the pre-COVID-19 group (30.2%). Furthermore, a smaller proportion of patients received pOP with PORT in the COVID-19 group (46.9%) compared to the pre-COVID-19 group (49.1%). Conversely, a higher proportion of patients received only pOP in the COVID-19 group (26.5%) compared to the pre-COVID-19 group (3.8%). In the pre-COVID-19 group, 9 patients (17.0%) received salvage surgery, compared to 5 patients (10.2%) in the COVID-19 group.

There was no significant difference in the type of surgery performed before and during the pandemic (*P* ═ 0.859).

### Survival

Next, we analyzed whether the COVID-19 pandemic had an impact on short-term survival. Univariable Cox regression analysis revealed no significant difference in OS (HR 0.84, 95% CI 0.27–2.66; *P* ═ 0.772) between patients treated prior to the COVID-19 pandemic compared to patients treated during the COVID-19 pandemic (Table 2). Patients with T3-4 tumors (HR 3.59, 95% CI 1.16–11.13; *P* ═ 0.027) and positive lymph nodes (HR 4.50, 95% CI 1.33–15.22; *P* ═ 0.015) had significantly poorer OS rates compared to those with T1-2 tumors and negative lymph nodes, respectively (Table 2). Moreover, patients who received pOP with PORT demonstrated significantly better OS rates (HR 0.19, 95% CI 0.04–0.96; *P* ═ 0.045) compared to patients receiving only pOP (Table 2).

**Table 2:** Univariable Cox regression analysis of patient demographic characteristics, tumor characteristics, types of surgery, and utilized treatment modalities

**Variables/Levels**	**OS**		
	*n*	**HR (95% CI)**	*P* **value**
*COVID-19 pandemic*	102		
COVID-19 vs pre-COVID-19 (ref)		0.84 (0.27 – 2.66)	0.773
*Age (years)*	102		
≥65 vs <65 (ref)		2.53 (0.76 – 8.41)	0.130
*Sex*	102		
Female vs male (ref)		2.35 (0.51 – 10.74)	0.271
*Initial diagnose*	102		
Yes vs no (ref)		0.36 (0.10 – 1.33)	0.125
*T stage*	102		
3-4 vs 1-2 (ref)		3.59 (1.16 – 11.13)	**0.027**
*N stage*	102		
+ vs 0 (ref)		4.50 (1.33 – 15.22)	**0.015**
*M stage*	102		
+ vs 0 (ref)		4.70 (0.61 – 36.52)	0.139
*Stage*	102		
III-IV vs I-II (ref)		3.07 (0.99 – 9.57)	0.052
*Grade*	85		
2 vs 1 (ref)		1.51 (0.19 – 11.80)	0.695
3 vs 1 (ref)		0.57 (0.04 – 9.10)	0.690
*Type of surgery*	102		
TL+ND vs TL (ref)		1.93 (0.35 – 10.58)	0.448
CE vs TL (ref)		0.32 (0.05 – 2.29)	0.258
STPL vs TL (ref)		4.34 (0.61 – 30.88)	0.142
SGPL vs TL (ref)		9.33 (1.22 – 71.51)	**0.032**
*Treatment modality*	102		
pOP + PORT vs pOP (ref)		0.19 (0.04 – 0.96)	**0.045**
pOP + PORCT vs pOP (ref)		0.53 (0.11 – 2.64)	0.440
Salvage surgery vs pOP (ref)		0.83 (0.17 – 4.10)	0.815

Significant results (*P* ≤ 0.05) are highlighted in bold print. OS: Overall survival; HR: Hazard ratio; COVID-19: Coronavirus disease 2019; TL: Total laryngectomy; ND: Neck dissection; CE: Cordectomy; STPL: Supratracheal partial laryngectomy; SGPL: Supraglottic partial laryngectomy; pOP: Primary surgery; PORT: Postoperative radiotherapy; PORCT: Postoperative radio-chemotherapy.

**Figure 1.**
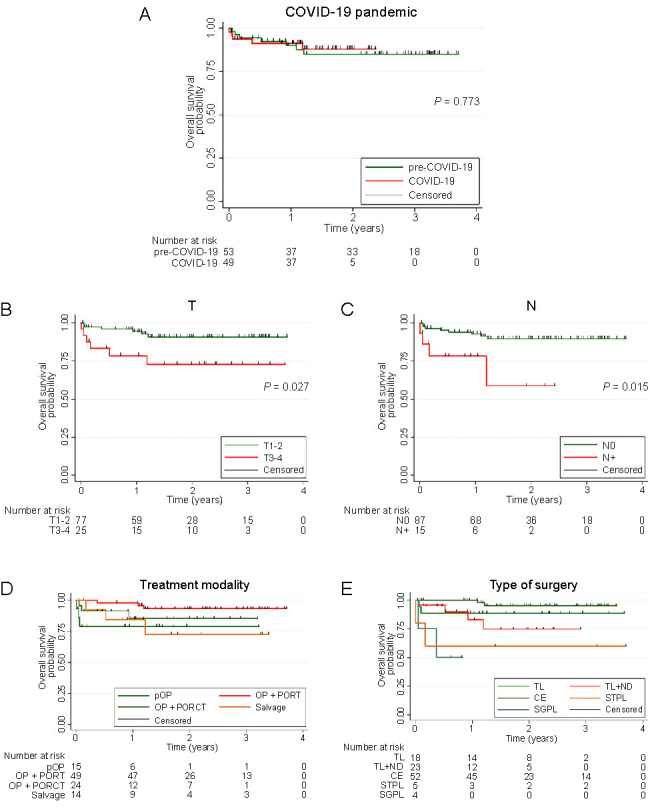
**The Kaplan–Meier plots and survival curves of OS.** (A) For patients treated pre-COVID-19 compared to those treated during the COVID-19 pandemic; (B) For T1-2 tumors compared to T3-4 tumors; (C) For patients with N0 lymph node status compared to patients with N+ lymph node status; (D) For treatment modalities (pOP, pOP with PORT, pOP with PORCT, and salvage surgery); (E) For the types of primary surgery (TL, TL with ND, CE, STPL, and SGPL). OS: Overall survival; COVID-19: Coronavirus disease 2019; pOP: Primary surgery; PORT: Postoperative radiotherapy; PORCT: Postoperative radio-chemotherapy; TL: Total laryngectomy; ND: Neck dissection; CE: Cordectomy; STPL: Supratracheal partial laryngectomy; SGPL: Supraglottic partial laryngectomy.

The Kaplan–Meier survival curves are depicted in Figure 1. The one-year survival rate for patients treated pre-COVID-19 was 90.1%, while it was 91.4% for those treated during the COVID-19 pandemic (Figure 1A). The one-year survival rate of T1-2 tumors stood at 94.5%, compared to 91.4% in T3-4 tumors (Figure 1B). Patients with N0 lymph node status had a one-year survival rate of 92.8%, whereas those with N+ lymph node status had a 78.3% one-year survival rate (Figure 1C). As for treatment modalities, the one-year survival rates were 79.0% for pOP, 98.0% for pOP with PORT, 85.2% for pOP with PORCT, and 84.6% for salvage surgery (Figure 1D). Lastly, by the type of primary surgery, the one-year survival rates were 88.9% for TL, 82.9% for TL with ND, 100% for CE, 60.0% for STPL, and 50.0% for SGPL (Figure 1E).

## Discussion

The current study provides first insights into the impact of the COVID-19 pandemic on the patient characteristics, treatment algorithms, and outcomes of surgically treated LSCC patients at our tertiary medical center. Indeed, we observed fewer patients undergoing adjuvant postoperative treatment during the pandemic. This reduction did not appear to negatively impact OS within our cohort. Other clinical and pathohistological characteristics did not differ before and during the pandemic.

Interestingly, our findings regarding patient demographics and tumor characteristics diverge from those of previous reports on head and neck cancer patients. The majority of studies have reported a COVID-19-related delay in therapy and an increased tumor burden [2, 13–15]. However, not every part of the world was equally affected by the pandemic. Studies from France and Italy did not report any increase in head and neck tumor burden [16, 17]. Additionally, existing literature on LSCC patient characteristics presents conflicting viewpoints. While Elibol et al. [18] found a significant increase in the proportion of T4-stage laryngeal tumors during the pandemic, Murri et al. [17] observed no such difference. These discrepancies in the literature might reflect the heterogeneity across individual countries, cities, and hospitals. Factors, such as initial COVID-19 prevalence, the extent of governmental restrictive measures, including movement restrictions and closures of public institutions, as well as existing referral networks and healthcare resources, all influenced how individual countries coped with the pandemic. Our results showed no tumor stage migration during the pandemic.

Importantly, our study revealed a significant difference in postoperative treatment modalities during the pandemic compared to pre-pandemic times. In particular, fewer patients underwent postoperative adjuvant treatment. To the best of our knowledge, we are the first group to report this trend for LSCC. Literature on adjuvant treatment modalities for head and neck cancer during the pandemic is limited. Solis et al. compared the characteristics of 137 head and neck cancer patients before and during the pandemic. Contrary to our findings, they observed significantly larger tumors in their COVID-19 group and no difference in adjuvant therapy [2]. Furthermore, the evidence regarding the impact of COVID-19 on treatment decisions for other types of cancer remains inconclusive. While some studies report a significant reduction in the use of adjuvant therapy [19], others indicate an increase in neoadjuvant therapy options [20]. The levels of evidence in these studies are not yet sufficient and do not yet allow to draw robust conclusions. Nevertheless, we believe it is reasonable to conclude that the COVID-19 pandemic has had an impact on treatment algorithms.

The reasons for the reduced utilization of adjuvant therapy during the COVID-19 pandemic could be multifaceted. Adjuvant therapy plays a crucial role in the treatment of head and neck cancer. It is typically used in higher stages but it may be considered in earlier stages of high-risk cases too [7]. However, both the oncologist’s assessment and the patient’s preferences play an equally important role in the decision-making process. This process may have been influenced by several factors. On the one hand, the notably low survival rates among COVID-19-infected cancer patients could have contributed to more cautious oncological decision making [21]. Additionally, expert recommendations and guidelines may also have discouraged the use of adjuvant therapy [22]. Ürün et al. investigated the decision-making practices of oncologists during the first month of the pandemic. Their findings revealed that 50% of oncologists reduced their usage of adjuvant therapy compared to pre-pandemic times, while 42% considered the administration of cytotoxic chemotherapy to be unsafe during the pandemic [23]. Furthermore, 80% of the oncologists reported that they would reduce the number of chemotherapy cycles administered [23]. Patient apprehension about hospital visits during the pandemic may have further contributed to decreased utilization of adjuvant therapy [24]. PORCT requires multiple visits to the oncology outpatient clinic, and during the pandemic patients may have avoided this therapy option to minimize the risk of COVID-19 transmission and infection [25]. A shift away from PORCT to alternative therapeutic modalities during the pandemic could have direct clinical consequences. For patients at risk, such as those with positive resection margins, extra nodal spread, and vascular invasion, PORCT has been shown to result in improved local control, prolonged progression-free survival, and increased OS compared to PORT [8].

The potential impact of the COVID-19 pandemic on long-term survival is a subject of ongoing research. In our study, we observed no significant difference in survival between patients treated before and during the COVID-19 pandemic. However, due to the very short follow-up period, a definitive conclusion cannot yet be drawn. To date, only one study has analyzed the potential impact of treatment changes during the COVID-19 pandemic on the survival of cancer patients. Venkatasai et al. examined 75 head and neck cancer patients who received primary radiotherapy prior to and during the pandemic. Their study indicated a trend toward worse survival for patients treated during COVID-19, despite similar treatment modalities [6]. These findings, along with ours, should be interpreted with caution. Depending on the primary site and stage, the 5-year survival rate for laryngeal cancer ranges between 78% and 35% [8]. If COVID-19-related limitations have an impact on cancer patient survival, such effect would likely not become evident for several years. Therefore, longer follow-up periods must be considered in future studies to obtain more reliable results. Nonetheless, it is important to closely monitor survival rates to detect any negative trends early on. Furthermore, a detailed analysis of therapy regimens, such as radiation intensity, chemotherapeutic dosages, and therapy intervals, is also critical for identifying variables that may affect patient survival. Our findings highlight the importance of continued monitoring and investigation into the impact of COVID-19 on cancer care.

Our study had several limitations that warrant consideration. First, the retrospective study design inherently carries biases, particularly regarding data availability, as it relies on the use of historical data. As a result, it may not adequately capture the full complexity of the patient population. Second, we only reported the tumor staging without precise localization, as this information was not properly recorded. Third, this study is based on the experience of a single institution, potentially limiting the generalizability of our findings. However, the data do reflect the specific needs of our local patient population. Fourth, our study cohort is relatively small, although it is comparable in size to other studies in this field [18, 26]. Larger studies are needed to validate our findings and provide more robust conclusions. Lastly, due to the limited follow-up period, our study only provides one-year survival data. Subsequently, longer follow-up periods will be necessary to assess whether the observed shifts in treatment modalities have any long-term impact on survival outcomes.

## Conclusion

In conclusion, our study provides initial evidence suggesting that the COVID-19 pandemic may have led to reduced utilization of postoperative adjuvant treatment in LSCC patients, which seemingly did not negatively impact the OS. Further research involving larger study cohorts and longer follow-up periods is warranted to validate our findings and to investigate the potential effects of such changes in treatment modalities on long-term survival.
